# Biosynthesis of Silver Nanoparticles Mediated by Entomopathogenic Fungi: Antimicrobial Resistance, Nanopesticides, and Toxicity

**DOI:** 10.3390/antibiotics10070852

**Published:** 2021-07-13

**Authors:** Tárcio S. Santos, Tarcisio M. Silva, Juliana C. Cardoso, Ricardo L. C. de Albuquerque-Júnior, Aleksandra Zielinska, Eliana B. Souto, Patrícia Severino, Marcelo da Costa Mendonça

**Affiliations:** 1 University of Tiradentes (Unit), Av. Murilo Dantas, Aracaju 49010-390, Brazil; tarcio.souza@souunit.com.br (T.S.S.); tarcisio.aragao@gmail.com (T.M.S.); juaracaju@yahoo.com.br (J.C.C.); ricardo_albuquerque@unit.br (R.L.C.d.A.-J.); patricia_severino@itp.org.br (P.S.); 2 Nanomedicine and Nanotechnology Laboratory (LNMed), Institute of Technology and Research (ITP), Av. Murilo Dantas, Aracaju 49010-390, Brazil; 3 Faculty of Pharmacy, University of Coimbra, Pólo das Ciências da Saúde, Azinhaga de Santa Comba, 3000-548 Coimbra, Portugal; zielinska-aleksandra@wp.pl; 4 Institute of Human Genetics, Polish Academy of Sciences, 60-479 Poznan, Poland; 5 CEB—Centre of Biological Engineering, Campus de Gualtar, University of Minho, 4710-057 Braga, Portugal; 6 Sergipe Agricultural Development Company (Emdagro), Av. Carlos Rodrigues da Cruz s/n, Aracaju 49081-015, Brazil

**Keywords:** entomopathogenic fungi, silver nanoparticles, biological synthesis, anti-microbials, insect control

## Abstract

Silver nanoparticles are widely used in the biomedical and agri-food fields due to their versatility. The use of biological methods for the synthesis of silver nanoparticles has increased considerably due to their feasibility and high biocompatibility. In general, microorganisms have been widely explored for the production of silver nanoparticles for several applications. The objective of this work was to evaluate the use of entomopathogenic fungi for the biological synthesis of silver nanoparticles, in comparison to the use of other filamentous fungi, and the possibility of using these nanoparticles as antimicrobial agents and for the control of insect pests. In addition, the in vitro methods commonly used to assess the toxicity of these materials are discussed. Several species of filamentous fungi are known to have the ability to form silver nanoparticles, but few studies have been conducted on the potential of entomopathogenic fungi to produce these materials. The investigation of the toxicity of silver nanoparticles is usually carried out in vitro through cytotoxicity/genotoxicity analyses, using well-established methodologies, such as MTT and comet assays, respectively. The use of silver nanoparticles obtained through entomopathogenic fungi against insects is mainly focused on mosquitoes that transmit diseases to humans, with satisfactory results regarding mortality estimates. Entomopathogenic fungi can be employed in the synthesis of silver nanoparticles for potential use in insect control, but there is a need to expand studies on toxicity so to enable their use also in insect control in agriculture.

## 1. Introduction

Nanotechnology has provided a new vision for the development of new processes and products. It can be applied for several purposes, such as to promote improvements in human and animal health, create more durable consumer goods, increase agricultural and industrial productivity [1,2]. As a result, nanoparticles have been used for different goals, as a method to deliver fertilizers, in the development of antimicrobials against bacterial resistance, as environmental monitoring biosensors, and for pest control [3,4,5,6].

Thus, metal and metal-oxide nanoparticles are being studied as materials with high potential for pest control [4,7,8]. Emphasis has been put on silver nanoparticles (AgNPs) for their easy synthesis, which can be performed by different methods and reducing/stabilizing agents, and versatility of application, due to their physical-chemical characteristics and toxicity on several insects [4,6,9,10,11]. Currently, biological synthesis has been extensively explored for the production of AgNPs because it is sustainable, with high reproducibility and low production cost. Several metabolites from plants and microorganisms can be used in this synthesis method, promoting simultaneously nanoparticles’ reduction and stabilization, as well as promoting the adhesion and formation of a layer of biomolecules on their surface, the corona, which increases their biocompatibility [12].

Filamentous fungi are microorganisms commonly used as mediators in biological synthesis methods of AgNPs because they produce and secrete high levels of metabolites, many of which have the ability to reduce metal salts and form nanoparticles, and can be handled easily. Several species of filamentous fungi have been used for the synthesis of AgNPs, mainly directed against microorganisms [13,14,15,16,17,18]. Despite the potential of these microorganisms for the synthesis of AgNPs, entomopathogenic filamentous fungi are still little used for this purpose. Their use is interesting for the development of nanoparticles for insect control, because these fungi produce enzymes and mycotoxins with an insecticidal effect and may have a satisfactory synergistic effect.

## 2. Silver Nanoparticles: General Features and Synthesis Approaches

The term “nanoparticle” refers to nanosized materials (usually between 0 and 100 nm) that have three dimensions (3D). The use of silver for the development of nanoparticles increased initially because of its biomedical applications, mainly as an effective antimicrobial agent [3,19]. Over the years, with the improvement of studies in relation to nanomaterials, it was possible to recognize several possibilities of application for silver nanoparticles (AgNPs), such in the development of food packaging to protect against microorganisms [20,21], the production of biosensors [22,23], and water decontamination [24,25].

**Figure 1:**
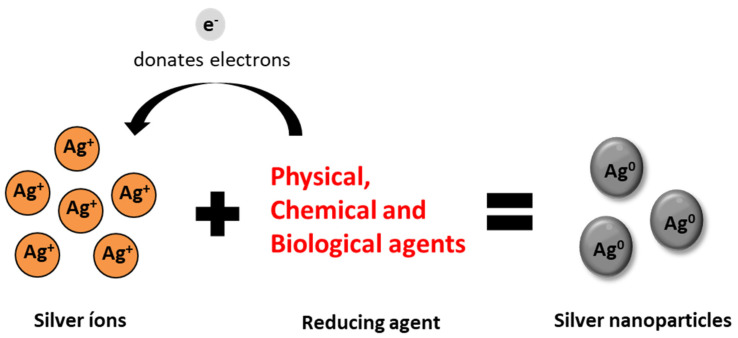
Schematic representation of the oxidation–reduction reaction in the formation of silver nanoparticles.

At the basis of the formation of AgNPs is the development of an oxidation reaction, where silver ions (Ag^+^) are reduced by interaction with a reducing agent and are transformed into neutral atoms (Ag^0^) (Figure 1). This reaction can be mediated by different reducing agents, through physical, chemical, or biological methods [26].

Physical methods, such as evaporation–condensation [27] and laser ablation [28], can be used for the synthesis of silver nanoparticles; however, the necessary equipment occupies large spaces, presents a high expenditure of electrical energy, and the yield in the final production is low, making its use for large-scale production disadvantageous. Chemical methods are the most traditional ones for the synthesis of silver nanoparticles and are based on chemical substances with a reducing action (such as sodium citrate, potassium bitartrate, and sodium borohydride) to promote the reduction of silver ions, the formation of nanoparticles, and the stabilization of active substances (such as humic acid, polyvinylpyrrolidone, alginate, chitosan) to prevent aggregation of the particles [19,29]. The chemical methods are effective and fast for the formation of silver nanoparticles; however, the use of reducing agents with high toxicity has implications for the subsequent use of the resulting nanoparticles, because chemical residues may remain in the nanomaterial even after its processing [30,31].

The methods of biological synthesis, also known as biosynthesis, use plant extracts, microorganisms, or biomolecules to reduce silver salts and convert them into silver nanoparticles. Different organic molecules can act as reducing agents in the oxidation–reduction reaction of silver ions, such as flavonoids, polyphenols, vitamins, proteins, terpenoids, and catechins that have the potential to reduce metal salts [12,32].

**Figure 2:**
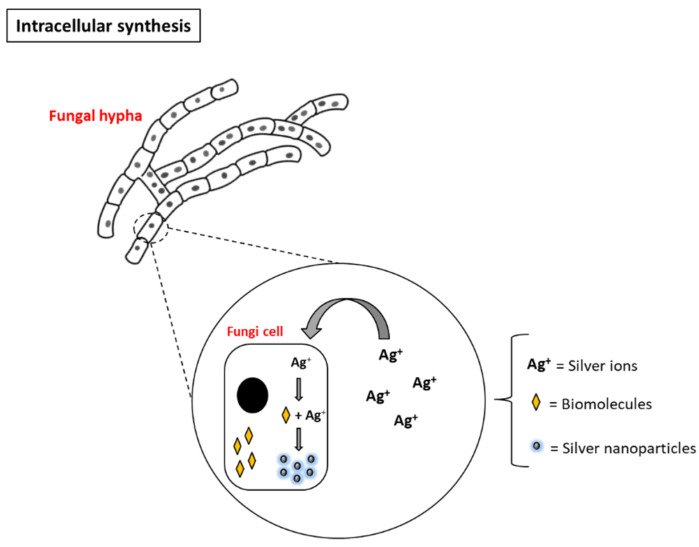
Intracellular biosynthesis of silver nanoparticles mediated by fungi.

Microorganisms, such as bacteria and filamentous fungi, are commonly used in the biosynthesis of silver nanoparticles. The synthesis mediated by these microorganisms can occur in the intracellular or extracellular medium, depending on the species used in the synthesis reaction. In intracellular synthesis, the formation of nanoparticle occurs inside the cells, because it is necessary that the microorganisms come into direct contact with the silver ions that will be absorbed and metabolized within the cells (Figure 2). The microorganisms that can perform this synthesis tend to form small and stable nanoparticles; however, additional steps are required to separate the nanoparticles from the cellular structures, which may not be advantageous [26,33].

**Figure 3:**
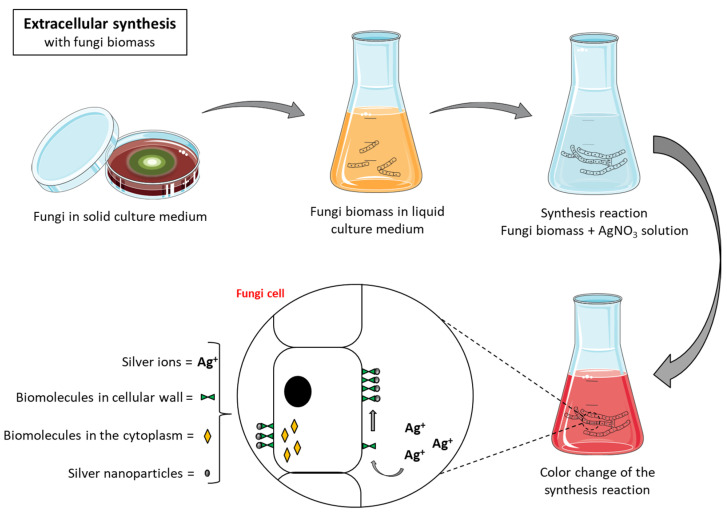
Extracellular biosynthesis of silver nanoparticles with fungal biomass.

Extracellular synthesis can involve either microbial biomass in direct contact with silver or extracts from microorganisms. Extracellular synthesis mediated by microbial biomass occurs through the interaction of silver ions with proteins present on the external surface of microorganisms’ cell wall, due to negative charges on the cell wall surface due to the presence of carboxylic groups or amine groups (Figure 3). The resulting nanoparticles adhere to the surface of the microorganisms, which makes it necessary to use separation techniques to isolate the nanoparticles, similar to what observed in intracellular synthesis, a factor that can generate more costs during material processing [34,35].

**Figure 4:**
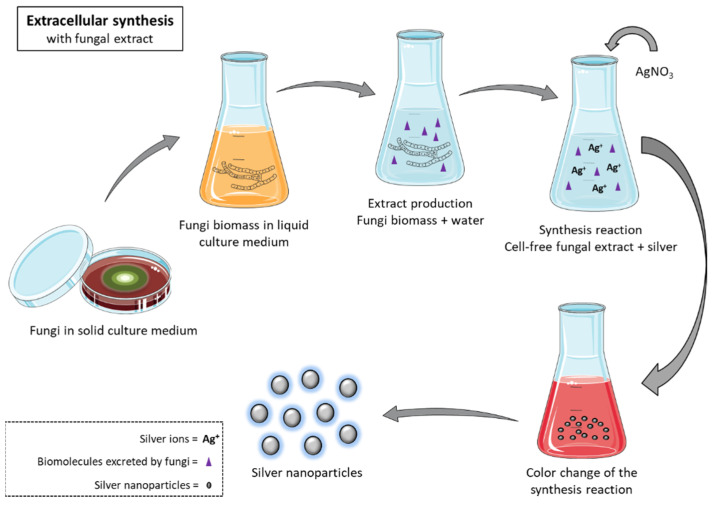
Extracellular biosynthesis of silver nanoparticles with cell-free fungal extract.

The synthesis of silver nanoparticles through aqueous extracts of microorganisms occurs through the action of biomolecules present in these extracts, derived from the biomass of the microorganisms (Figure 4). Thus, the nanoparticles form in a medium free of undesirable components. This is the most used method for the synthesis of silver nanoparticles using microorganisms [34].

The mechanism of production of silver nanoparticles by bacteria and fungi varies depending on the species of microorganism or strain used. The production and release of NADH-dependent reductases are involved in the extracellular synthesis of silver nanoparticles mediated by some fungi and bacteria, such as *Fusarium oxysporum*; however, not all fungi use this enzyme. Other biomolecules produced by these microorganisms, such as co-enzymes, naphthoquinones, and anthraquinines, can also participate in the reduction of silver. Therefore, different microorganisms can interact differently with a given metal, depending on their metabolism. The complete mechanism of synthesis of silver nanoparticles by microorganisms is still not fully elucidated [32,36].

The main advantage of biological synthesis for the production of silver nanoparticles is its ecological character. This synthesis method allows the use of biodegradable or innocuous substances, reducing the possibility of causing environmental damage. Therefore, it is considered an environmentally sustainable method. Another advantage is linked to the stabilization of the resulting particles. In biological synthesis, the production and stabilization of nanoparticles occur at the same time, since the reducing agent, in addition to synthesizing the nanoparticles, can also participate in the stabilization of the nanoparticles, a process that in other synthesis methods requires the addition of specific substances. This occurs because the biomolecules present in the reaction medium are adsorbed on the surface of the nanoparticles, forming an outer layer called the corona, which promotes the stabilization of the particles and can also improve their biological action and/or biocompatibility [37].

A disadvantage of biological synthesis is the possibility of a difference in the concentrations of the biomolecules present in the biological source (whether microorganisms or plants) due to variations in the environment or in the availability of nutrients. Thus, there is a need to use rigorous methodological processes, monitoring the effect of factors such as pH, temperature, stirring speed, silver concentration, and amount of fungal biomass in during the synthesis reaction, in order to minimize possible interferences and optimize the formation of nanoparticles [38,39,40]. Despite this, silver nanoparticles synthesized by biological routes have potential for use in several areas. The search for new agents with reducing capacity and new applications is boosting this area of study, favoring its understanding and development.

## 3. Biological Synthesis of Silver Nanoparticles with Entomopathogenic Fungi, Application against Drug-Resistant Bacteria and in Insect Control

Entomopathogenic fungi (EF) are microorganisms known to use insects as a temporary host during their life cycle, a characteristic that justified their study and manipulation as biological agents for pest control. These microorganisms participate in the natural regulation of insect populations in the environment and in the decomposition cycle of organic matter [41,42]. Entomopathogenic fungi have great phylogenetic diversity and include approximately 700 species, distributed in the phyla Oomycota, Chytridiomycota, Microsporidia, Entomophtoromycota, Basidiomycota, e Ascomycota [43].

Entomopathogenic fungi have a global distribution, with greater abundance in tropical zones, and can be found in the soil or colonizing the body of dead arthropods. They are classified according to their morphology into single-celled or multicellular filaments and, as to their way of obtaining nutrients, in heterotrophic, comprising entomophages (referring to the species that colonize live arthropods, triggering diseases and possibly leading to their death), and saprophages (species that colonize the body of dead arthropods) [41].

The mechanism of infection of entomopathogenic fungi in their host occurs through physical and chemical interactions and is initiated with the contact between the spore of the fungus and the integument of the host. In this phase, called adhesion phase, the colonization site is recognized, and the spore is fixed on the host’s integument by the action of proteins (adhesins). Subsequently, the penetration phase begins, with the metabolization and development of penetration hyphae, having an appressorium at its tip, whose function is to break the host integument by mechanical action, for the subsequent infection. The penetration of the fungus is also mediated by the secretion of enzymes (mostly proteases, lipases, and chitinases) that aid in the degradation of the integument and facilitate the mechanical action of the appressorium. Upon entering the host’s body, the colonization phase begins, where the fungus spreads inside the host through the release of hyphal bodies in the hemolymph, colonizing the internal organs and causing the insect’s death. At the end of this process, the fungus hyphae colonize the exterior of the host’s body, and new spores are produced and disseminated in the environment [44].

The use of entomopathogenic fungi in the control of insect pests is widely explored. There are different types of commercial products based on entomopathogenic fungi, with *Beauveria bassiana* and *Metarhizium ani*s*opliae* being the most used species for the development of commercial formulations due to their broad spectrum of action and the ability to infect several species of insects at all stages of their development. Despite this, their use in insect control has limitations. As they are living organisms, abiotic factors (temperature, humidity, radiation, luminosity) can modulate their metabolism and interfere with spores’ ability to germinate, which changes their infectious potential when applied in the field. In addition, these factors also affect the shelf life of these microorganisms when they are produced on a large scale for their commercialization, reducing the useful life of conidia (spores) when stored at room temperature [45]. Therefore, different methodologies have been developed to improve the use of these microorganisms in insect control, such as the use of entomopathogenic fungus in combination with vegetable oils [46], the supplementation of substances that promote greater resistance against harmful environmental factors, such as riboflavin and chitin [47,48], and spore encapsulation techniques [49,50].

**Figure 5:**
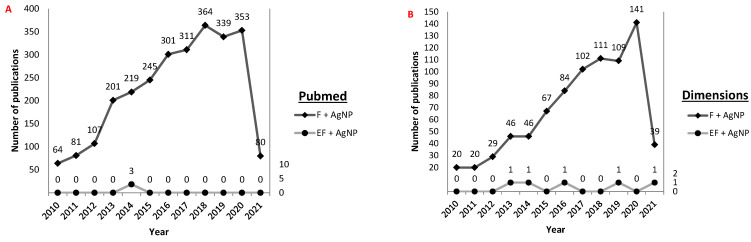
Prospecting the number of publications existing in scientific articles bases between the years 2010 and 2021: (**A**) = Pubmed, (**B**) = Dimensions. Keywords used for title and abstract search: “*fungi and silver nanoparticle*” (F + AgNP), “*entomopathogenic fungi and silver nanoparticle*” (EF + AgNP). The 2021 data refer only to the first three months of that year.

Thus, the application of the metabolites of entomopathogenic fungi for the development of silver nanoparticles is an alternative to the use of entomopathogenic fungi, though still little explored, as can indicated by the low number of articles indexed in scientific databases on this topic (Figure 5). The number of publications indicates a growth in the production of information about silver fungi and nanoparticles between the years 2010 and 2021; however, studies that relate entomopathogenic fungi to silver nanoparticles are scarce.

The first study on the application of entomopathogenic fungi for the synthesis of silver nanoparticles dates from 2013, and since then, few studies showing the efficiency of the use of these microorganisms for the synthesis of silver nanoparticles and their possible applications have been published (Table 1). *B. bassiana*, *M. anisopliae*, *I. fumosorosea*, and *Trichoderma harzianum* are the species of entomopathogenic fungi with potential for the synthesis of silver nanoparticles, through extracellular synthesis based on aqueous extracts of the fungi. In addition, silver nanoparticles synthesized with extracts of entomopathogenic fungi have differences in size (ranging from 10 and 200 nm) and in morphology (spheres, triangles, hexagons, or tubes) (Table 1).

The mechanism of formation of the nanoparticles mediated by entomopathogenic fungi is not yet fully understood. There is a possibility that the formation of the silver nanoparticles by entomopathogenic fungi requires nitrate reductases, as observed for other filamentous fungi such as *Aspergillus spp*. [51] and *Fusarium oxysporum* [52], with NADPH^+^ (nicotinamide adenine dinucleotide phosphate) enzymes as the reaction cofactors. However, there may be variation in the production of biomolecules between species and different fungi isolates, requiring direct assessments to identify components related to the formation of the silver nanoparticles.

**Table 1:** Records of the use of entomopathogenic fungi in the biosynthesis of silver nanoparticles, with the method of synthesis used, diameter and morphology of the nanoparticles formed, and bacteria and target insects.

Fungus Species	Method of Synthesis	Diameter & Nanoparticle Morphology	Target Bacteria	Target Insect	Reference
*Beauveria bassiana*	Extracellular	36.88–60.93 nm, spherical	–	*Aedes aegypti* (Diptera: Culicidae)	[53]
	Extracellular	20.44–34.16 nm, spherical	*Escherichia coli* and *Staphylococcus aureus*	*Aedes aegypti*, *Anopheles stephensi* and *Culex quinquefasciatus* (Diptera: Culicidae)	[54]
	Extracellular	10–50 nm, spherical, triangular, hexagonal	*Escherichia coli*, *Pseudomonas aeruginosa* and *Staphylococcus aureus*	–	[55]
	Extracellular	40.14–289.13 nm	–	–	[56]
*Metarizhium anisopliae*	Extracellular	132.3 nm	–	–	[56]
*Isaria fumosorosea*	Extracellular	51.31–111.02 nm, spherical	–	*Aedes aegypti* and *Culex quinquefasciatus* (Diptera: Culicidae)	[57]
	Extracellular	131.3 nm	–	–	[56]
*Trichoderma harzianum*	Extracellular	10–20 nm, irregular form	–	*Aedes aegypti* (Diptera: Culicidae)	[30]

The antibacterial application of silver nanoparticles synthesized from entomopathogenic fungi has been focused on bacteria with resistance to drugs such as *Escherichia coli*, *Staphylococcus aureus,* and *Pseudomonas aeruginosa* (Table 1) [54,55]. The effectiveness of these particles in reducing bacterial development is related to the interaction between the characteristics of the nanoparticles (mainly, its size, shape, and surface electrical charge) and bacterial morphological structures. Electrical charges on the surface of the bacterial cell wall can repel silver nanoparticles, thus reducing their effectiveness [55]. Therefore, these parameters must be carefully evaluated for this type of application.

Assessments of the insecticidal effect of silver nanoparticles synthesized with entomopathogenic fungi have been carried out mainly against urban pests and vectors of human pathogens, such as *Aedes aegypti* mosquitoes (dengue, zika, and chikungunya vector), *Anopheles stephensi* (malaria vector), and *Culex quinquefasciatus* (lymphatic filariasis vector) (Diptera: Culicidae) (Table 1) [30,53,54,57]. No studies have been found on the application of AgNPs synthesized with entomopathogenic fungi for the control of agricultural pests in the indexing bases of publications; however, in general, studies have already shown the potential of biogenic silver nanoparticles for the control of insects that cause damage to agricultural crops, such as S*podoptera littoralis* (Lepidoptera: Noctuidae) [58], *Spodoptera litura* (Lepidoptera: Noctuidae) [59], *Amritodus brevistylus* (Hemiptera; Cicadellidae) (Shanmugapriya et al., 2017), *Plutella xylostella* (Lepidoptera: Plutellidae) [60], *Planococcus citri* (Hemiptera: Pseudococcidae) [61], *Helicoverpa armigera* (Lepidoptera: Noctuidae) [62].

Silver nanoparticles synthesized with entomopathogenic fungi may thus be an alternative for pest control in agricultural crops; however, further studies need to be carried out in order to close the existing gap on information about this type of particle, as to evidence if there is a synergistic relationship between the fungi metabolites and silver in the action against insects and how it can be manipulated for use in agriculture. In addition, a greater understanding of the toxicity of these materials in insects and non-target organisms and their impact on the environment is necessary to favor their use in agriculture [63,64]. In addition, it is necessary to develop methodologies to acquire more information about the toxicity of these nanoparticles in order to guarantee safety in their application.

## 4. Toxicity Assessment of Silver Nanoparticles

In general, the toxicity of a substance may be related to its chemical composition, concentration, and interaction with target organisms. Silver nanoparticles are small in size and have a large interaction surface with the external environment; therefore, information about their toxicity is important to demonstrate their effectiveness and possibility of use, as well as the safety of their application [65].

In vitro toxicity assessment methodologies have been used for the characterization of silver nanoparticles, presenting low cost and allowing a quick and efficient analysis. The speed of these techniques allows them to be used to improve the physical and chemical properties of nanoparticles before increasing the scale of their production or carrying out more complex analyses. In vitro analysis methods can be used to assess the toxicity of silver nanoparticles on cells and nucleic acids (cytotoxicity and genotoxicity) and on plants (phytotoxicity) [66,67].

The impact caused by AgNPs on cells can be observed through several approaches, such as by assessing the influence of nanoparticles on the development of morphological changes in cells (through phase-contrast microscopy) and physiological processes (production of reactive oxygen species—ROS—evaluation cell proliferation, change in mitochondrial metabolism) [68,69]. The MTT assay is one of the most widely used test to assess eukaryotic cell cytotoxicity using laboratory cell cultures. The technique consists in the evaluation of the mitochondrial metabolism of cells by observing the reduction of 3- (4,5-dimethylthiazol-2)-2,5-diphenyltetrazolium bromide, also known as tetrazolium bromide, thiazolyl blue, or MTT, producing formazan. MTT has a yellow color, while formazan, resulting from its reduction, has a purple color. The change in the color of the reaction can be evaluated through a spectrophotometer using visible light, and cell viability is expressed by comparison to untreated cells [66].

The MTT assay is widely used for the analysis of cytotoxicity caused by silver nanoparticles [70,71], and has demonstrated that the cytotoxic effect depends directly on the physical-chemical characteristics of the nanoparticles, their concentration, the exposure time, and the target cell.

The International Organization for Standardization (ISO) recommends the use of genotoxicity tests to evaluate products based on nanomaterials, in order to assess the safety of their application. According to ISO, in vitro and in vivo analysis methodologies can be used to assess genotoxicity, with a primary focus on mammalian cells, with in vitro methods being the most used, in particular, the micronucleus test and the comet assay, which are easy to perform and provide reliable results [72].

The comet assay allows observing the integrity of DNA right after its exposure to a toxic agent and can be used for analysis of prokaryotic and eukaryotic cells [73,74]. Due to its simple methodology, it is widely used in toxicological studies and is applied to analyses of AgNPs in animal and plant cells [75]. The micronucleus test, based on blocking cytokinesis, reveals the presence of fragmented genetic material in cells, which tends to cluster in small portions at the margin of the cell nucleus, proving the occurrence of mutagenesis. This test can be used by itself or in association with other tests, such as the comet assay [76,77].

Genotoxicity assays for AgNPs can also be performed by evaluating the direct effect of nanoparticles on plant cells. For this, onion (*Allium cepa*) is the indicator organism most used for this type of analysis because it is considered a sensitive model to detect substances capable of promoting chromosomal abnormalities. The technique consists of exposing the roots of the plant to the agent to be analyzed, in different concentrations and for a determined time, and then the meristematic cells of the roots are analyzed and quantified under an optical microscope to detect possible abnormalities [24,78].

The Amest Test is another methodology for the analysis of genotoxicity in vitro that can be applied for the evaluation of AgNPs. This method uses a genetically modified *Salmonella typhimurium* bacterium to observe the percentage of genotoxicity through the analysis of mutation induction [79].

Considering that silver nanoparticles can be used for the control of insects affecting agricultural crops, evaluations of the phytotoxic and insecticidal effect of nanoparticles are also necessary to identify the concentration threshold that promotes pest control without causing damage to the plants. Phytotoxicity tests evaluate the influence of substances on the development of plants and can be carried out by observing the development of the seed (germination, root growth, and stem) or the appearance of deformities in the leaves, over the time of exposure to the treatment [80,81].

Different plant species can be used as model organisms to study the phytotoxicity of silver nanoparticles, such as zucchini [82], tomato [83,84], castor [85], oats, lettuce, and radish [86]. In general, methodologies using lettuce (*Lactuca sativa*) as a model in phytotoxicity tests are used for the analysis of several substances, as they are simple and fast, of low cost, and with good results, since the lettuce is sensitive to the presence of elements that promote environmental stress [87].

Bioassays for evaluating the insecticidal effect of substances are commonly performed in order to determine their efficiency and feasibility of use, with an initial focus on analysis in laboratory conditions, whose results can then be extrapolated to field conditions. This type of evaluation can be carried out through different methodologies, targeting different species of insects, and the method to be used in this evaluation is directly related to the target insect of the investigation.

In insect pathology, it is common to use some species of insects as alternative models for evaluating insecticidal action, in order to make initial observations about the insecticidal character of a substance or product before extrapolating the bioassays to more complex organisms. *Galleria mellonella* (Lepidoptera: Pyralidae), *Drosophila melanogaster* (Diptera: Drosophilidae), *Bombyx mori* (Lepidoptera: Bombycidae), and *Tenebrio molitor* (Coleoptera: Tenebrionidae) [12,88] are commonly used as analysis models for the insecticidal action of substances and pathogens, but there are no reports in the literature about a particular species of insect that is considered an alternative model for assessing the insecticidal potential of nanoparticles.

Among these species of model insects, *G. mellonella*, *D. melanogaster*, and *T. molitor* were used to analyze the toxicity of silver nanoparticles; *T. molitor* that is easy to create and handle, a characteristic that also facilitates its use for this purpose [12,89,90]. The choice of insect species must be made considering the feasibility of their use as toxicity models or the existence of phylogenetic proximity with other insect species of economic importance.

## 5. Conclusions

This review article discusses the general aspects of silver nanoparticles, as well as the use of biological synthesis for their production, with emphasis on the use of entomopathogenic fungi, their antibacterial and pest control applications, and the importance of toxicity analysis. The biological synthesis of silver nanoparticles is effective and sustainable. In this context, the use of entomopathogenic fungi for the biosynthesis of silver nanoparticles is still little explored, which highlights the need for further studies on this topic. Silver nanoparticles synthesized with extracts of these microorganisms have potential for application in several areas as antimicrobial agents or for insect pest control. They can be an alternative product for the management of agricultural pests, provided that tests are carried out to ensure their effectiveness and the lack of damage to non-target organisms, humans, and the environment. The limited information available so far is the main factor limiting their use.

## Data Availability

Data are available from corresponding authors upon request.
